# ADAMTS-4_v1 Is a Splice Variant of ADAMTS-4 That Is Expressed as a Protein in Human Synovium and Cleaves Aggrecan at the Interglobular Domain

**DOI:** 10.1002/art.38102

**Published:** 2013-10-28

**Authors:** Shane D Wainwright, Jan Bondeson, Bruce Caterson, Clare E Hughes

**Affiliations:** Shane D. Wainwright, PhD, Jan Bondeson, MD, PhD, Bruce Caterson, PhD, Clare E. Hughes, PhD: Cardiff UniversityCardiff, UK

## Abstract

**Objective:**

We previously described a messenger RNA variant of *ADAMTS4* (*ADAMTS4_v1*) in human synovial cell cocultures obtained from patients with osteoarthritis (OA). This RNA message has been found only in OA synovium and, if translated, would result in a protein identical to ADAMTS-4, except that the C-terminal spacer domain would be different. The purpose of this study was to determine whether *ADAMTS4_v1* is translated into a protein, is expressed in vivo, and acts as a functional aggrecanase.

**Methods:**

Polyclonal antibodies were raised against unique C-terminal sequences of ADAMTS-4_v1. An immunohistochemical study of human OA synovium was performed. A mammalian expression vector coding for FLAG-tagged human *ADAMTS4* was mutated to contain the different sequences of *ADAMTS4_v1*, and the resultant plasmid was used to transfect HEK 293 cells. ADAMTS-4_v1 produced by these cells was purified via the FLAG epitope, and the ability of this recombinant protein to cleave aggrecan, biglycan, and decorin was investigated.

**Results:**

An antibody specific for ADAMTS-4_v1 was found to bind to the synovial membrane surface on cryosections, and the protein was detected in cell lysates from synovium obtained from OA patients. The recombinant ADAMTS-4_v1 demonstrated enzyme activity toward the target substrate in a commercial aggrecanase 1 enzyme-linked immunosorbent assay and was also found to cleave aggrecan at the pathologically important Glu^373↓374^Ala aggrecanase site.

**Conclusion:**

ADAMTS-4_v1 is expressed as a protein in vivo in human OA synovium, functions as an aggrecanase, and cleaves other proteoglycan substrates. This splice variant may be a major contributor to loss of aggrecan from the superficial zone of OA cartilage.

Osteoarthritis (OA) is a multifaceted disease that involves pathologic changes in the articular cartilage, subchondral bone, surrounding soft tissue, and synovium (1,2). Inflammation and proliferation of the synovium are reported in both the early and late stages of OA (3,4). Synovial tissue characteristically becomes thickened, and higher synovial fluid volumes are correlated with progression of OA, joint space narrowing, and bone marrow lesions (1). Inflamed OA synovial tissue is known to release a plethora of cytokines and proteinases capable of degrading nearby cartilage and other connective tissues.

OA synovium produces several members of the ADAMTS family of metalloproteinases, including ADAMTS-4 and ADAMTS-5, key enzymes involved in the cleavage of aggrecan and the degradation of cartilage (5–7). Aggrecan degradation and loss from cartilage is an early and crucial event in the development of OA, contributing to the loss of function and the development of joint pain that are manifested in this disease. Contributions made by ADAMTS-4 and ADAMTS-5 in the progression of human OA are ambiguous, with studies showing involvement of one or both of these enzymes (8–11). We have previously reported that culture of a mixed population (coculture) of human synovial cells derived from OA synovium express messenger RNA (mRNA) that encodes for a splice variant of ADAMTS-4, with the potential of producing a protein with a C-terminal spacer domain sharing no homologies with the ADAMTS-4 spacer domain (12). This mRNA has so far been found only in OA synovium and has not been detected in any other joint tissue, including OA cartilage. The resultant splice-variant protein is predicted to retain enzyme specificity for aggrecan degradation, although properties dependent on the ADAMTS-4 spacer domain would be lost.

In the present study, we describe the production and characterization of this splice variant, named ADAMTS-4_v1, as a recombinant protein. We produced antibodies to its C-terminal domain and showed that it is expressed as a protein in synovial tissue from OA patients, and we investigated its ability to cleave aggrecan in the interglobular domain (IGD) and degrade other matrix proteoglycans.

## Materials and Methods

### Construction of *ADAMTS4_v1*-pCEP4 plasmid

*ADAMTS4.1*-pCEP4 containing the complementary DNA of human *ADAMTS4* 3′ FLAG in the pCEP4 plasmid (Invitrogen) was a gift from Hideaki Nagase (Imperial College London, London, UK). The 162 basepairs from the 5′ end of exon 9 were removed using a QuikChange XL site-directed mutagenesis kit (Agilent Technologies), with primers named CEH310 (5′-GTCAGGCTCCTTCAGGAAATTCAGATGTGGTACTGCCTGGGGCAGTCAGC-3′) and CEH311 (5′-GCTGACTGCCCCAGGCAGTACCACATCTGAATTTCCTGAAGGAGCCTGAC-3′). The product CEH310/311-pCEP4 lacked the sequence between the stop codons of *ADAMTS4* and *ADAMTS4_v1*. The missing sequence was inserted using the unique *Bst* ZI restriction enzyme site in exon 9 of *ADAMTS4*. CEH310/311 was cut out and ligated into pBluescript (Agilent Technologies), and the sequence lying 3′ of the *Bst* ZI site was removed. The 3′ end of *ADAMTS4_v1* was constructed by reverse transcription–polymerase chain reaction (RT-PCR) of RNA extracted from OA synovial cells, as described previously (12), using an Advantage 2 PCR kit (BD Biosciences) with primers named CEH319 (5′-CACACGCCTCCGATACAGCTTCTTC-3′) and CEH320 (5′-GGCAGTTTAGATGGAGGGCTGTCTG-3′). The FLAG sequence was added using sequential PCR with the primers named CEH319 and CEH321 (5′-ATCGTCATCTTTATAATCCTGCCTCCCAGGGCAGGAAACCAG-3′), followed by primers CEH319 and CEH322 (5′-GAGCTCGAGTTACTTGTCATCGTCATCTTTATAATCCTGCCT-3′). The product CEH319/322 was ligated into *Bst* ZI/*Sac* I–cut CEH310/311-pBluescript. The complete sequence of *ADAMTS4_v1* was cut from pBluescript using *Kpn* I and *Xho* I and then ligated into pCEP4. The *ADAMTS4_v1*-pCEP4 was subjected to DNA sequencing using in-house facilities.

### Production of recombinant proteins

*ADAMTS4.1*-pCEP4 and *ADAMTS4_v1*-pCEP4 were stably transfected into HEK 293 cells maintained in Dulbecco's modified Eagle's medium (DMEM) containing 250 μg/ml of G418, 1× antibiotic/antimycotic solution, and 10% fetal bovine serum (FBS; Invitrogen) using FuGene 6 (Roche Diagnostics) according to the manufacturer's protocol. After 48 hours, the cells were transferred to medium supplemented with 200 μg/ml of hygromycin B (Invitrogen) for selection. Cells were maintained in this medium and were harvested and the medium was replenished every 3 days.

### Purification of FLAG-labeled recombinant proteins by immunoprecipitation

Cells were harvested by centrifugation at 400*g*, washed in phosphate buffered saline (Dulbecco A solution; Oxoid), and incubated for 30 minutes at 4°C in lysis buffer (1% Triton X-100, 50 m*M* Tris HCl, pH 7.4, 1 m*M* EDTA, 150 m*M* NaCl, and Halt protease inhibitor cocktail [Thermo Scientific]). The lysate was cleared by centrifugation at 12,000*g* for 10 minutes at 4°C. Conditioned medium was precleared by centrifugation at 400*g* and filtered using a 0.22-μm Stericup/Steritop filter unit (Millipore). The cell lysate or conditioned medium was incubated for 4 hours at 4°C with 40 μl of anti–FLAG M2 affinity gel (Sigma). The gel was washed in lysis buffer and Tris buffered saline (TBS; 50 m*M* Tris HCl, pH 7.4, 150 m*M* NaCl), and bound proteins were eluted by incubation with 150 ng/μl of 3× FLAG peptide (Sigma) in TBS for 30 minutes at 4°C. Eluted proteins were recovered by centrifugation, filtered (0.22-μm Ultrafree-MC filter units [Millipore]), and stored at −80°C.

### Production of antibodies to the C-terminus of ADAMTS-4_v1

Sequences ^735^PGHTPPIQLLRAPADP^750^ (AltTS4.1) and ^805^RELLLLPHAKTQWGGAVGVRP^825^ (AltTS4.2) of ADAMTS-4_v1 were selected using the EMBOSS software program (13) and synthesized on multiantigenic peptide cores and controlled-pore glass (Alta Bioscience). Rabbits were immunized subcutaneously (500 μg per injection) with either the AltTS4.1 or the AltTS4.2 ADAMTS-4_v1 sequences in Freund's complete adjuvant and were given 4 booster injections in Freund's incomplete adjuvant at 28-day intervals. Animals were bled 14 days after each booster immunization. Antibodies were affinity-purified on peptide-coated controlled-pore glass according to the manufacturer's protocol.

### Sodium dodecyl sulfate–polyacrylamide gel electrophoresis (SDS-PAGE) and Western blotting

Samples were subjected to SDS-PAGE using either 10% or 4–12% gels (Invitrogen) and transferred to Protran nitrocellulose membranes (Whatman) using a Bio-Rad Mini Trans-Blot cell. Samples were then blocked with 5% (weight/volume) bovine serum albumin (BSA) in TSA (50 m*M* Tris HCl, pH 7.4, 200 m*M* NaCl, 0.02% [w/v] sodium azide) and probed with antibody in 1% (w/v) BSA in TSA, followed by alkaline phosphatase–conjugated anti-mouse IgG (Promega) or anti-rabbit IgG (Dako). Color was developed with BCIP/nitroblue tetrazolium substrate (Promega). Protein bands were visualized with Coomassie brilliant blue R250. Primary antibodies ab28285 and ab39201 were from Abcam and BAF4307 from R&D Systems.

### Immunohistochemistry

Synovium was obtained from 2 patients undergoing knee replacement surgery for OA (ethical consent was obtained according to South East Wales Local Research Ethics Committees directive DEBP/el/03-5102). Samples were embedded in OCT (Fisher Scientific) using liquid nitrogen-cooled isopentane, and 10-μm tissue sections were cut, air-dried, and fixed with ice-cold 90% ethanol. The sections were washed in 0.1% Tween 20 in PBS, blocked for 20 minutes with 2.5% horse serum in PBS, and incubated for 20 minutes in 2.5% horse serum with the primary antibody. Binding was detected with a Ready-to-Use Vectastain kit (Vector), visualized with the use of a Vector NovaRED kit, counterstained with Mayer's hemalum, dehydrated, and mounted in DPX mountant.

### Isolation and culture of synovial cells

Synovium from 2 patients with OA was trimmed of fat, diced, and digested for 2 hours at 37°C with 1 mg/ml of collagenase I and DNase in DMEM containing 10% FBS (14). The suspension was filtered through a 40-μm cell strainer. Cells were plated at 2 × 10^6^ /ml and cultured for 48 hours prior to solubilization in lysis buffer for SDS-PAGE and Western blotting.

### Aggrecanase 1/ADAMTS-4 assay

A SensoLyte 520 aggrecanase 1 assay kit (AnaSpec) was used to detect aggrecanase 1/ADAMTS-4 activity. Truncated human ADAMTS-4 (0.251 pmoles) in 50 μl of component C, 50 μl of purified ADAMTS-4_v1, or 50 μl of furin-activated ADAMTS-4_v1 was added to the wells of a black Sera-Wel 96-well microtiter plate (Sterilin). Diluted aggrecanase substrate buffer (50 μl) was added, and the plate was incubated at 37°C for 1 hour in a FluoStar Optima microplate instrument (BMG Lab Technologies), with monitoring at 490 nm/520 nm excitation/emission spectra and readings obtained every 5 minutes.

### Aggrecanase assay

Bovine aggrecan (20 μg; Sigma) was incubated overnight at 37°C in the presence or absence of 2 units of furin (New England Biolabs), with purified ADAMTS-4, ADAMTS-4_v1, or anti-FLAG immunoprecipitates from cell lysates of untransfected HEK 293 cells, in TBS containing 10 m*M* CaCl_2_. Digests were deglycosylated with chondroitinase ABC (Sigma), keratanase, and keratanase II (Seikagaku Kogyo) and then subjected to Western blotting with the aggrecan IGD aggrecanase site-specific neoepitope monoclonal antibody (mAb) BC-3 (15).

### Digestion of small leucine-rich proteoglycans by ADAMTS-4 and ADAMTS-4_v1

Bovine nasal cartilage was extracted and subjected to associative density-gradient ultracentrifugation as previously described (16). The A3 extract (25 μg per digestion) was incubated overnight at 37°C with either purified ADAMTS-4 or ADAMTS-4_v1 or with anti-FLAG immunoprecipitates from cell lysates of untransfected HEK 293 cells, in TBS containing 10 m*M* CaCl_2_. Digests were deglycosylated (15) and subjected to Western blotting using either the biglycan-specific mAb PR-1 (17) or the decorin-specific mAb 70.6 (18).

## Results

### Production and characterization of ADAMTS-4_v1

**Figure 1:**
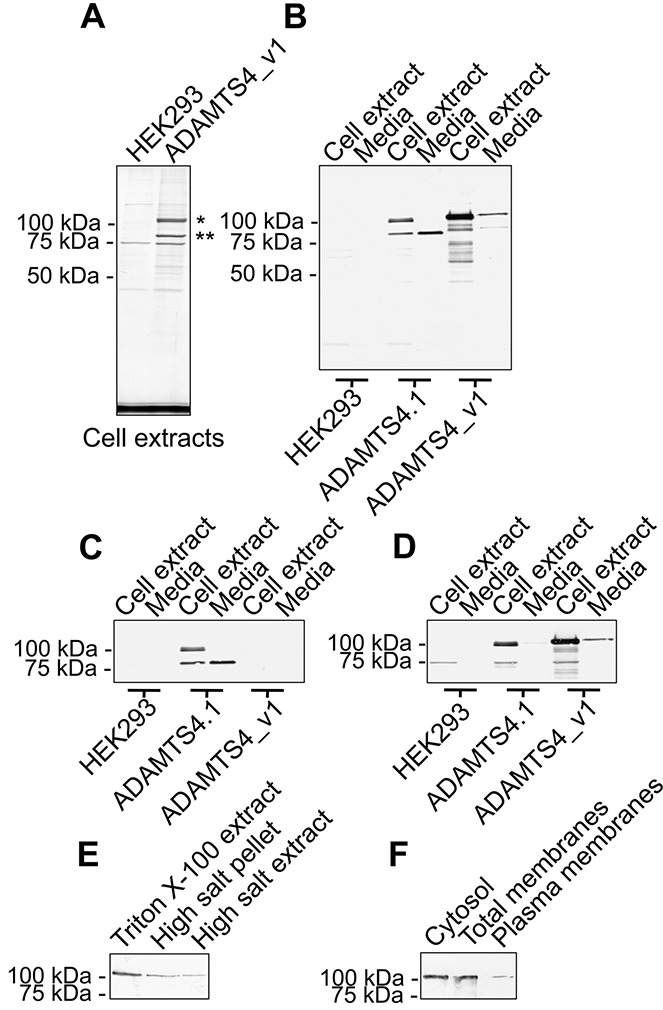
Characterization of recombinant ADAMTS-4_v1. A, Blot of anti–FLAG M2 monoclonal antibody (mAb) immunoprecipitates from Triton X-100 extracts of untransfected HEK 293 cells and *ADAMTS4_v1*-pCEP4–transfected HEK 293 cells, following sodium dodecyl sulfate–polyacrylamide gel electrophoresis and Coomassie brilliant blue R250 staining. The band marked with an asterisk yielded an N-terminal amino acid sequence of ASPLPRE…, whereas no sequence was obtained from the band marked with a double asterisk. B–D, Triplicate Western blots of anti–FLAG M2 mAb immunoprecipitates from Triton X-100 cell extracts and conditioned media obtained from untransfected, *ADAMTS4.1*-pCEP4–transfected, or *ADAMTS4_v1*-pCEP4–transfected HEK 293 cells, probed with anti–FLAG M2 mAb (B), a rabbit antiserum (ab28285) to the carboxyl-terminus of ADAMTS-4 (C), or a rabbit antiserum (ab39201) to the pro domain of ADAMTS-4 (D). E, Western blot of anti–FLAG M2 mAb immunoprecipitates from a Triton X-100 extract of an equal number of Tris buffered saline–washed *ADAMTS4_v1*-pCEP4–transfected HEK 293 cells (Triton X-100 extract), a Triton X-100 extract of the pellet after high salt extraction (high salt pellet), and a 1*M* NaCl, 50 m*M* Tris HCl, pH 7.4, extract of *ADAMTS4_v1*-pCEP4–transfected HEK 293 cells (high salt extract). F, Western blot probed with anti–FLAG M2 mAb using a plasma membrane protein extraction kit to prepare cytosol, total membranes, and plasma membranes from *ADAMTS4_v1*-pCEP4–transfected HEK 293 cells prior to anti–FLAG M2 mAb immunoprecipitation.

The splice variant plasmid was used to stably transfect HEK 293 cells. A Coomassie brilliant blue R250–stained SDS-PAGE gel of anti-FLAG immunoprecipitates from detergent lysates of *ADAMTS4_v1*-pCEP4–transfected HEK 293 cells showed 2 prominent bands at 104 and 82 kd (Figure 1A) that were absent in immunoprecipitates from untransfected cells. Protein sequence analysis of the 104-kd band (Figure 1A) revealed ASPLPRE…, corresponding to the predicted N-terminus for the recombinant band, while no sequence was obtained from the 82-kd band (Figure 1A). These bands match closely with the predicted molecular weight of 91 kd and 69 kd for the full-length and furin-activated ADAMTS-4_v1 proteins.

To further characterize these proteins, we performed a series of Western blots on purified ADAMTS-4_v1 and ADAMTS-4 from either cell extracts or conditioned medium. A Western blot of purified ADAMTS-4_v1 from cell extracts and media (Figure 1B) that was probed with the anti–FLAG M2 mAb, showed a prominent band of 104 kd and a weaker band of 82 kd, confirming that these bands represented 2 different forms of ADAMTS-4_v1. Further FLAG-containing bands of 88, 67, and 48 kd were seen in the immunoprecipitate from the cell extracts (Figure 1B) and may represent lower molecular weight breakdown products of ADAMTS-4_v1. The apparent 104 and 82 kd for ADAMTS-4_v1 were slightly slower migrating than bands detected in purified ADAMTS-4 from cell extracts and media (Figure 1B).

The 104- and 82-kd bands of ADAMTS-4_v1 did not label with an antibody to the carboxyl-terminus of ADAMTS-4 (Figure 1C); however, as expected, this antibody did label ADAMTS-4 proteins of 95 kd and 70 kd (Figure 1C). In contrast, an antibody reacting with the pro domain of ADAMTS-4 labeled only the 104-kd ADAMTS-4_v1 protein and the 95-kd ADAMTS-4 protein (Figure 1D). Interestingly, this antibody also detected a 104-kd band in ADAMTS-4_v1 purified from the conditioned media (Figure 1D). A 70-kd cross-reactive band (as reported by Abcam) was detected in all cell extracts (Figure 1D). Taken together, these data show that *ADAMTS4_v1*-pCEP4–transfected HEK 293 cells produced the full-length ADAMTS-4_v1 protein and an 82-kd form minus its pro domain; both forms were found in the conditioned media.

To determine whether ADAMTS-4_v1 is secreted as a peripheral membrane protein, a high salt extract of ADAMTS-4_v1-transfected HEK 293 cells was prepared and analyzed by Western blotting (Figure 1E). Interestingly, the proenzyme was extracted from the cells using high salt. Further analysis of cytosol, total membranes, and plasma membranes from these cells showed the presence of proenzyme in all of these cellular extracts (Figure 1F).

### Production and characterization of antibodies to the C-terminal domain of ADAMTS-4_v1

**Figure 2:**
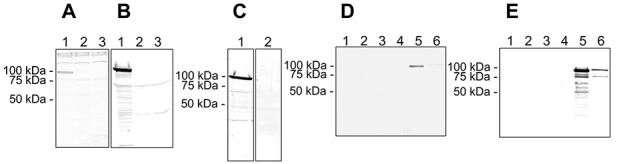
Characterization of antisera raised against the ADAMTS-4_v1 synthetic peptides. A and B, Western blots of Triton X-100 extracts of HEK 293 cells transfected with *ADAMTS4_v1*-pCEP4 (lane 1), *ADAMTS4.1*-pCEP4 (lane 2), or left untransfected (lane 3) and probed with the AltTS4.1 serum (A) or the AltTS4.2 serum (B). C, Western blot of a Triton X-100 extract of HEK 293 cells transfected with *ADAMTS4_v1*-pCEP4 and probed with affinity-purified AltTS4.2 antibodies (lane 1) or with affinity-purified AltTS4.2 antibodies that had been preincubated with 5 μg/ml of the AltTS4.2 peptide (lane 2). D and E, Duplicate Western blots of anti-FLAG immunoprecipitates from Triton X-100 cell extracts (lanes 1, 3, and 5) and conditioned media (lanes 2, 4, and 6) from untransfected HEK 293 cells (lanes 1 and 2), HEK 293 transfected with *ADAMTS4.1*-pCEP4 (lanes 3 and 4), or HEK 293 cells transfected with the *ADAMTS4_v1*-pCEP4 (lanes 5 and 6) and then probed with AltTS4.1 serum (D) or AltTS4.2 serum (E).

Polyclonal antibodies specific to ADAMTS-4_v1, named AltTS4.1 and AltTS4.2, were used to probe Western blots of both synthetic peptides. It was found that the antibodies labeled only their corresponding peptide, with no evidence of cross-reactivity (data not shown). Western blots were performed on detergent lysates from untransfected HEK 293 cells or from HEK 293 cells producing either ADAMTS-4_v1 or ADAMTS-4. Both antibodies labeled a 104-kd band in the ADAMTS-4_v1 lysate (Figures 2A and B), with no reactivity with the ADAMTS-4 lysate (Figures 2A and B) or with the lysate from untransfected cells (Figures 2A and B).

AltTS4.1 appeared to have a lower affinity for the 104-kd band and have some cross-reactivity with higher molecular weight bands in all the lysates (Figure 2A). Identical Western blots of a detergent lysate of ADAMTS-4_v1 were probed with affinity-purified AltTS4.2 antibodies preincubated in the presence or absence of the immunizing peptide AltTS4.2. As expected, the 104-kd band labeled with AltTS4.2, and this labeling was abrogated by preincubation with the immunizing peptide, thus confirming the specificity of this antibody. To further confirm specificity, Western blots were performed on ADAMTS-4 and ADAMTS-4_v1 purified by anti-FLAG immunoprecipitation from detergent lysates and conditioned media. Antibody AltTS4.1 detected a 104-kd band in ADAMTS-4_v1 purified from lysates (Figure 2D), and staining was barely detectable in ADAMTS-4_v1 purified from conditioned medium (Figure 2D). In contrast, antibody AltTS4.2 reacted with 104- and 82-kd bands in ADAMTS-4_v1 purified from conditioned medium (Figure 2E) and cell lysates (Figure 2E), and these appeared similar to bands detected on duplicate Western blots probed with the anti–FLAG M2 mAb (Figure 1B). Additional bands were labeled by AltTS4.2 in ADAMTS-4_v1 purified from cell lysates (Figure 2E) and appeared similar to those seen in an identical Western blot probed with the anti–FLAG M2 mAb (Figure 2B). No reactivity of either the AltTS4.1 or the AltTS4.2 antibody was seen with purified ADAMTS-4 (Figures 2D and E) or with anti-FLAG immunoprecipitates from untransfected HEK 293 cells (Figures 2D and E).

### In vivo protein expression of ADAMTS-4_v1

**Figure 3:**
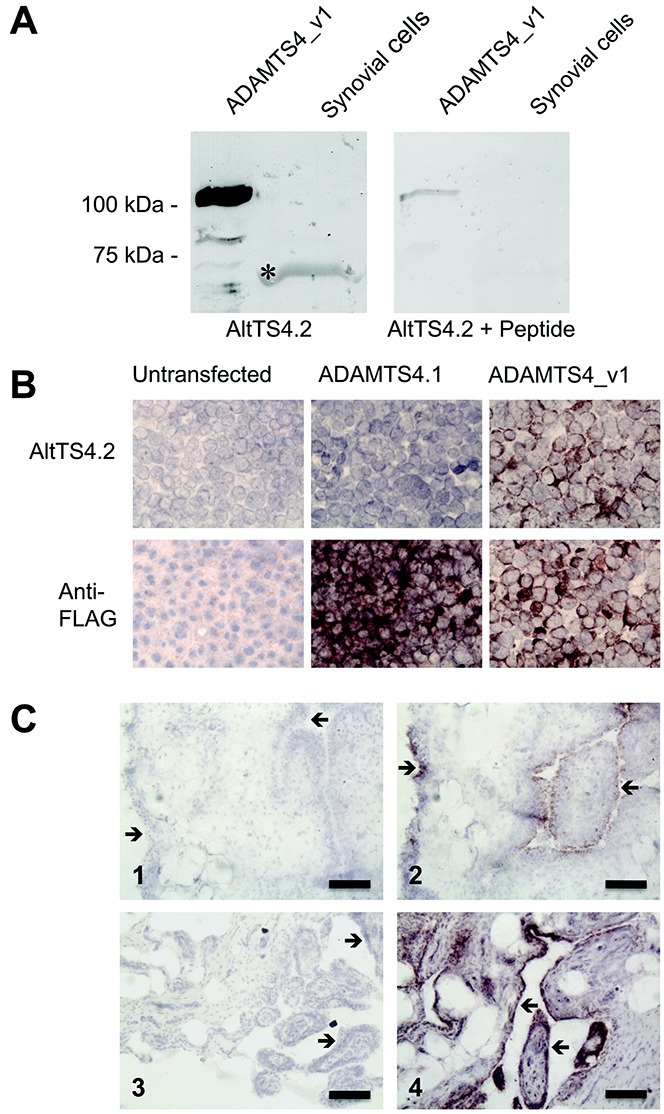
Analysis of synovial cells and synovium from patients with osteoarthritis (OA) using the ADAMTS-4_v1 specific antibody AltTS4.2. A, Western blots of a mixed cell population (coculture) isolated from OA synovium. Purified recombinant ADAMTS-4_v1 and detergent lysates of OA synovial cocultures were subjected to sodium dodecyl sulfate–polyacrylamide gel electrophoresis and Western blot analysis. Blots were probed with affinity-purified AltTS4.2 that had been raised against a synthetic peptide within the unique C-terminus of ADAMTS-4_v1 or were probed with AltTS4.2 that had been preincubated with 5 μg/ml of the immunizing peptide. The asterisk indicates the band that stained with the AltTS4.2 antibody in synovial cell lysates. B, Immunohistochemical analysis of AltTS4.2 antibodies. Monolayers of untransfected HEK 293 cells or HEK 293 cells transfected with *ADAMTS4.1*-pCEP4 or *ADAMTS4_v1*-pCEP4 were probed with affinity-purified AltTS4.2 antibodies or the anti–FLAG M2 monoclonal antibody. Original magnification × 100. C, Immunohistochemical analysis of OA knee synovium using AltTS4.2 antibodies. Cryosections of knee synovium from OA patient A (1 and 2) and OA patient B (3 and 4) were incubated in the absence (1 and 3) or presence (2 and 4) of affinity-purified AltTS4.2. Sections were counterstained with Mayer's hemalum. Arrows indicate the synovial membrane and villi surfaces. Bars = 100 μm.

In vivo protein expression of ADAMTS-4_v1 in synovial tissue was investigated by Western blotting of short- term cultures of OA synovial cells and immunohistochemistry of OA synovial tissue (Figures 3A and C). Western blotting with AltTS4.2 of cells isolated from human OA synovium (Figure 3A) showed positive staining of a band migrating with a molecular weight of ∼70 kd. The specificity of this band was confirmed by peptide inhibition (Figure 3A), where there was no staining for this band after preincubation of AltTS4 with the immunizing peptide.

Monolayers of untransfected or transfected (*ADAMTS4_v1*-pCEP4 and *ADAMTS4.1*-pCEP4) HEK 293 cells were probed with the anti–FLAG M2 mAb or with AltTS4.2 antibodies (Figure 3B). Untransfected cells showed no staining. Strong staining of the transfected cells was observed with the anti–FLAG M2 mAb. No staining with AltTS4.2 was seen in cells transfected with *ADAMTS4.1*-pCEP4, but strong staining was seen on cells transfected with *ADAMTS4_v1*-pCEP4. Cryosections of synovium from the knees of 2 patients with OA were probed with AltTS4.2 and showed strong staining in many areas of the synovial surface and villi in both patients (Figure 3C). Staining was particularly localized to the outer layer of cells in the villi of the synovium. Underlying tissue was generally negative, although occasional clusters of immunopositive cells were observed. Tissue sections incubated without primary antibody (Figure 3C) showed no staining.

### Proteolysis of ADAMTS-4_v1

**Figure 4:**
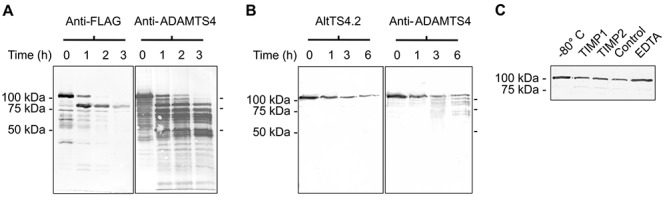
Proteolysis of ADAMTS-4_v1. A, Western blots probed with anti–FLAG M2 monoclonal antibody (mAb) or affinity-purified polyclonal goat antibodies to human ADAMTS-4 from purified ADAMTS-4_v1 that had been stored at −80°C during the course of the experiment (lane 0) or had been incubated with 2 units of furin at 37°C for 1, 2, or 3 hours (lanes 1, 2, and 3, respectively). B, Western blots probed with affinity-purified AltTS4.2, a rabbit antibody raised against a synthetic peptide representing a unique sequence in the C-terminus of ADAMTS-4_v1, or with affinity-purified goat antibodies to human ADAMTS-4 from purified ADAMTS-4_v1 that had been stored at −80°C during the course of the experiment (lane 0) or had been incubated at 37°C in the presence of 10 m*M* CaCl_2_ for 1, 3, or 6 hours (lanes 1, 3, and 6, respectively). C, Western blot probed with anti–FLAG M2 mAb from purified ADAMTS-4_v1 that had been stored at −80°C during the course of the experiment (−80°C) or had been incubated for 6 hours at 37°C in the presence of 10 m*M* CaCl_2_ (control) or 10 m*M* CaCl_2_ plus 24 n*M* tissue inhibitor of metalloproteinases 1 (TIMP-1), 23 n*M* TIMP-2, or 5 m*M* EDTA.

Removal of the pro domain by furin and/or autocatalysis of ADAMTS-4 results in activation of this enzyme (19). ADAMTS-4_v1 was incubated with furin for up to 3 hours at 37°C and monitored for catalysis by Western blotting using anti-FLAG and goat anti-human ADAMTS-4 antibodies (R&D Systems) (Figure 4). Western blotting showed decreased staining of the 104-kd band and a corresponding increased staining of an 82-kd band, showing that ADAMTS-4_v1 was cleaved by furin (Figure 4A). Furthermore, the intensity of staining of the 82-kd band at 3 hours appeared to be decreased in comparison to staining intensity at 1 hour and 2 hours. Western blotting with an antibody raised against the whole ADAMTS-4 protein (Figure 4A) revealed multiple catabolites of ADAMTS-4_v1 that increased over the 3 hours. As these bands are not detected by the C-terminal anti-FLAG, this suggests that the loss of the 82-kd band is most likely due to autocatalytic C-terminal processing of ADAMTS-4_v1, as has been reported to occur with ADAMTS-4 (20,21).

As autocatalysis has been reported for ADAMTS-4 (19–21), we incubated ADAMTS-4_v1 at 37°C for up to 6 hours (Figure 4B). Western blotting with antibody AltTS4.2 and anti-FLAG (data not shown) showed a decrease in the intensity of staining of the 104-kd band but no generation of an 82-kd band. However, Western blotting with anti–ADAMTS-4 showed multiple catabolites that were not detected by the C-terminal–domain antibodies. Furthermore, the loss of the 104-kd band was inhibited in the presence of 5 m*M* EDTA (Figure 4C) and was not greatly inhibited in the presence of tissue inhibitor of metalloproteinases 1 (TIMP-1; 24 n*M*) or TIMP-2 (23 n*M*). CaCl_2_ also modulated this C-terminal processing, as in its absence, the enzyme remained as a 104-kd band (data not shown). Collectively, these data indicate that the loss of the 104-kd band is due to autolytic processing either by the 104-kd protein or by an undetectable amount of the 82-kd protein present in these preparations.

### Enzyme substrate specificity of ADAMTS-4_v1

**Figure 5:**
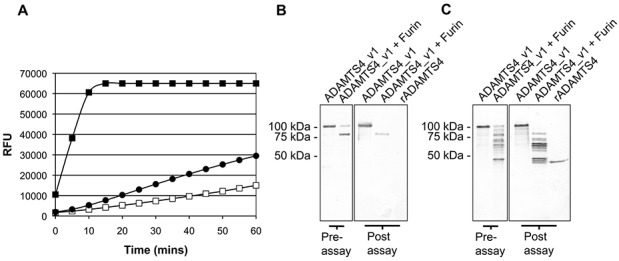
Aggrecanase substrate activity of ADAMTS-4_v1, as assessed by SensoLyte 520 aggrecanase 1 assay. A, Aggrecanase 1 activity of an anti–FLAG M2 immunoprecipitate from ADAMTS-4_v1 transfected with HEK 293 cells that had been predigested for 1 hour at 37°C with furin (▪) or an equal amount of undigested ADAMTS-4_v1 immunoprecipitate (□), as compared to that of the recombinant truncated ADAMTS-4 (•) (supplied in the assay kit). Enzyme activity (expressed as relative fluorescence units [RFU]) was measured every 5 minutes for 1 hour. B, Western blot probed with anti–FLAG M2 monoclonal antibody from anti–FLAG M2 immunoprecipitates from ADAMTS-4_v1–transfected HEK 293 cells used in A. Shown are undigested and furin-digested ADAMTS-4_v1 prior to their use in the assay (preassay) as well as undigested and furin-digested ADAMTS-4_v1 plus recombinant truncated ADAMTS-4 (rADAMTS-4) that was harvested from the assay plate after the 1-hour course of the aggrecanase 1 assay (postassay). C, Duplicate Western blot of B probed with affinity-purified goat anti-human ADAMTS-4 antibodies (BAF4307).

To determine whether ADAMTS-4_v1 is capable of cleaving aggrecan at the IGD cleavage site, we used a commercially available aggrecanase 1 assay kit that included a synthetic peptide aggrecanase 1 substrate. The aggrecanase 1 activity of purified full-length ADAMTS-4_v1 was compared to furin-activated ADAMTS-4_v1 and a truncated form of ADAMTS-4 (residues 213–579 of the full-length human ADAMTS-4). Preincubation of ADAMTS-4_v1 showed the highest rate of increase in relative fluorescence units (RFU), reaching saturation after ∼10 minutes, and was ∼20–25-fold greater than that of the full-length ADAMTS-4_v1 (Figure 5A). Western blot analysis prior to assay showed the efficiency of furin to generate the 82-kd form of the enzyme, and postassay analysis demonstrated that the 82-kd form was further processed during the 1-hour assay period (Figures 5B and C).

**Figure 6:**
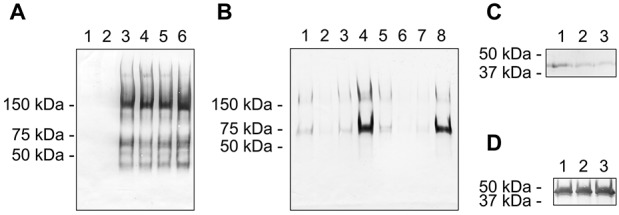
Enzyme activity of ADAMTS-4_v1 against proteoglycan substrates. A, Western blot of bovine aggrecan digested with anti-FLAG immunoprecipitates from Triton X-100 extracts of untransfected HEK 293 cells (lanes 1 and 2), purified ADAMTS-4.1 (lanes 3 and 4), and ADAMTS-4_v1 (lanes 5 and 6), in the absence (lanes 1, 3, and 5) or presence (lanes 2, 4, and 6) of furin. The blot was probed with neoepitope monoclonal antibody (mAb) BC-3 to detect cleavage at the interglobular domain aggrecanase cleavage site. B, Western blot of aggrecan (lanes 4 and 8) and aggrecan that had been deglycosylated with chondroitinase ABC (lanes 1 and 5), chondroitinase ABC plus keratanase I and keratanase II (lanes 2 and 6), or keratanase I plus keratanase II (lanes 3 and 7) digested with either ADAMTS-4.1 (lanes 1–4) or ADAMTS-4_v1 (lanes 5–8). Cleavage at the interglobular domain aggrecanase site was detected with BC-3. C and D, Western blots of an A3 extract of cartilage digested with anti-FLAG immunoprecipitates from Triton X-100 extracts of untransfected HEK 293 cells (lane 1), purified ADAMTS-4.1 (lane 2), or ADAMTS-4_v1 (lane 3) and then probed with mAb PR-1, which is specific for biglycan (C), or mAb 70.6, which is specific for decorin (D).

To estimate the molar concentrations of active ADAMTS-4_v1, densitometric analysis was performed. The initial rate of digest of the recombinant truncated form of ADAMTS-4 and active ADAMTS-4_v1 was calculated as 1,996 and 22,559 RFU/minute/pmole, respectively, indicating that ADAMTS-4_v1 was 10 times more active than the truncated form. However, this initial rate of reaction for ADAMTS-4_v1 is representative of multiple C-terminally truncated forms. To determine if ADAMTS-4_v1 could cleave native aggrecan at the IGD site (EGE…ARG), aggrecan was incubated with purified ADAMTS-4_v1 in the presence or absence of furin to activate the enzyme during the course of the experiment (Figure 6A). Cleavage at the IGD site was detected by Western blotting using the neoepitope mAb BC-3 (anti-ARG…). As shown in Figure 6A, ADAMTS-4_v1 could generate the BC-3 epitope in the presence and absence of furin. The increased activity generated by furin-activated versus full-length ADAMTS-4_v1, as illustrated in Figure 5A, was not observed when aggrecan was used as the substrate, possibly due to differences in the experimental conditions. In this experiment, the enzyme was not preactivated with furin, and an overnight digestion allowed ample time for autoactivation of the enzyme in the absence of furin. Detection of BC-3–positive bands ranging from 200 kd to 37 kd indicated cleavage at C-terminal sites by both enzymes (Figure 6A). After overnight incubation, the amount of BC-3 epitope generated and the fragment pattern obtained by ADAMTS-4_v1 appears to be equivalent to that generated by ADAMTS-4 (Figure 6A, with or without furin).

To determine whether the glycosaminoglycan requirements of ADAMTS-4_v1 digestion of aggrecan are the same as those reported for ADAMTS-4 (22), native aggrecan or aggrecan that had been predigested with chondroitinase ABC, with chondroitinase ABC, keratanase, and keratanase II, or with keratanase and keratanase II was digested with either purified ADAMTS-4 or ADAMTS-4_v1 and subjected to Western blotting using monoclonal antibody BC-3 (Figure 6B). Removal of chondroitin sulfate or keratan sulfate glycosaminoglycans greatly reduced the cleavage of aggrecan at the IGD site by both ADAMTS-4 and ADAMTS-4_v1 (Figure 6B). With both enzymes, it appears that removal of the keratan sulfate chains had a greater effect on aggrecan cleavage at the IGD site than did removal of the chondroitin sulfate chains (Figure 6B). These results suggest that the presence of keratan sulfate glycosaminoglycans facilitates IGD cleavage in aggrecan.

It has been reported that ADAMTS-4 is able to cleave other proteoglycans (23–26). We thus tested whether ADAMTS-4_v1 is capable of cleaving cartilage biglycan and decorin using the A3 fraction from bovine articular cartilage extracts as an enriched source of biglycan and decorin. Digestions of the A3 fraction were carried out with purified ADAMTS-4_v1 or ADAMTS-4. A digest of aggrecan was carried out to confirm enzyme activity (data not shown). The biglycan-specific antibody PR-1 labeled intact biglycan (37 kd) in A3 extracts that had been incubated with immunoprecipitates from untransfected HEK 293 cells (Figure 6C). A weak but noticeable reduction of biglycan labeling was seen in the A3 extracts digested with either ADAMTS-4 or ADAMTS-4_v1 (Figure 6C), although no lower molecular weight proteolytic fragments could be detected with this mAb. Antibody 70.6 labeled intact decorin in the A3 extracts incubated with immunoprecipitates from untransfected HEK 293 cells, and there was no apparent difference in the labeling seen when A3 was digested with purified ADAMTS-4_v1 or ADAMTS-4 (Figure 6D). No discernible proteolytic fragments could be detected with this mAb in any of the digests.

## Discussion

We have previously shown (12) that OA synovial cells produce an mRNA that codes for a splice variant of *ADAMTS4* (*ADAMTS4_v1*), producing a protein nearly identical to ADAMTS-4 in which the spacer domain is replaced with a nonhomologous C-terminal domain. Herein, we have clearly shown that the splice variant *ADAMTS4_v1* is translated as a protein in human OA synovium and synovial cells and that a purified recombinant ADAMTS-4_v1 is capable of cleaving aggrecan at the pathological IGD aggrecanase site.

Previous studies have shown that recombinant ADAMTS-4 is produced as a full-length protein in a number of cell lines and is furin-activated, resulting in removal of the pro domain prior to secretion as lower molecular weight forms (19,21,26). The major protein purified from ADAMTS-4_v1 cell lysates was the pro domain 104-kd protein, along with small amounts of the N-terminally processed (presumed to be furin-activated) 82-kd protein. Similar pro domain (95-kd) and furin-activated (70-kd) products were purified from ADAMTS-4 cell lysates. However, the only furin-activated (70-kd) form of ADAMTS-4 was detected in conditioned media, but surprisingly, the zymogen form of ADAMTS-4_v1 was the predominant species in the media from cell transfected with this plasmid. The presence of the pro domain form in the media may be due to cell death, or in contrast to ADAMTS-4, it may be that ADAMTS-4_v1 is secreted from HEK 293 cells into the media mainly as the pro domain form. Analysis of high salt extracts and plasma membrane preparations strongly indicate that the variant is secreted as a zymogen that associates with the cell membrane; however, whether this is the case in vivo is still to be determined. As reported for ADAMTS-4 (19), we found that ADAMTS-4_v1 is cleaved by the proprotein convertase furin. We have shown that ADAMTS-4_v1 is susceptible to autocatalysis, which is inhibited by EDTA, but not by TIMP-1 or TIMP-2. It seems likely that the ADAMTS-4_v1 secreted with the pro domain attached can be activated by extracellular pro domain convertase (27,28), by self-activation, or by some other unknown mechanism.

The sites of C-terminal cleavage for ADAMTS-4_v1 may be similar to those reported in the literature, where C-terminal processing of ADAMTS-4 involves cleavage in the spacer domain at Lys^694^–Phe^695^ to produce a 53-kd form and at Thr^581^–Phe^582^ in the cysteine-rich domain to produce a 40-kd form of ADAMTS-4, by the action of membrane type 4 matrix metalloproteinase (at the cell membrane) or by autolysis (20,29). The Thr^581^–Phe^582^ cleavage site is present on ADAMTS-4_v1, and although the Lys^694^–Phe^695^ bond is conserved in ADAMTS-4_v1, the sequence following the Arg^696^ diverges from that of ADAMTS-4, and this cleavage site therefore may not be efficiently recognized. In our studies using furin digestion of ADAMTS-4_v1 (Figures 4A and 5C), we observed extensive C-terminal processing. In summary, it can be speculated that the C- terminal domain may play a role in the overall stability, processing, and substrate specificity of ADAMTS-4_v1.

Our work clearly showed that ADAMTS-4_v1 could cleave aggrecan at the IGD site in the aggrecan core protein and other C-terminal sites, confirming that the spacer domain does not play a crucial role in cleavage at these sites. We also investigated the potential catabolism of decorin and biglycan. In those studies, we observed loss of immunolabeling of biglycan digested with either ADAMTS-4 or ADAMTS-4_v1, suggesting that there was some catalytic activity against this small leucine-rich proteoglycan. However, no digestion of decorin was observed with either enzyme, and this finding is in contrast to other published work on ADAMTS-4 specificity (26). Our source of decorin was a partially purified preparation, and so, perhaps there were other more favorable substrates within this preparation.

Antibodies to the C-terminal domain of ADAMTS- 4_v1 showed positive staining in OA synovium, confirming our mRNA data from synovial cell cocultures and the translation of this message into protein in vivo. The staining was most prominent in the superficial layer of the synovial membrane, which are areas associated with inflammation (30). In addition, we showed that isolated OA synovial cells produced a 70–75 kd ADAMTS-4_v1 protein. Taken together, these data suggest that ADAMTS-4_v1 is produced in vivo and may play a prominent role in the pathologic changes in OA cartilage.

In conclusion, we have shown that the previously described *ADAMTS4_v1* mRNA (12) is translated into a protein and that the recombinant protein is capable of digesting both aggrecan and biglycan, but not decorin. Importantly, we have localized this splice variant within the OA synovial membrane, and we speculate that this splice variant may play a role in aggrecan catabolism in the superficial zone of articular cartilage with the onset of OA.
